# Comparative Proteomics Reveal Me31B’s Interactome Dynamics, Expression Regulation, and Assembly Mechanism into Germ Granules during *Drosophila* Germline Development

**DOI:** 10.1038/s41598-020-57492-y

**Published:** 2020-01-17

**Authors:** Aidan McCambridge, Dhruv Solanki, Nicholas Olchawa, Neal Govani, Jonathan C. Trinidad, Ming Gao

**Affiliations:** 1 Biology Department, Indiana University Northwest, Gary, IN USA; 2 Department of Chemistry, Indiana University, Bloomington, IN USA

**Keywords:** Oogenesis, Protein-protein interaction networks

## Abstract

Me31B is a protein component of *Drosophila* germ granules and plays an important role in germline development by interacting with other proteins and RNAs. To understand the dynamic changes that the Me31B interactome undergoes from oogenesis to early embryogenesis, we characterized the early embryo Me31B interactome and compared it to the known ovary interactome. The two interactomes shared RNA regulation proteins, glycolytic enzymes, and cytoskeleton/motor proteins, but the core germ plasm proteins Vas, Tud, and Aub were significantly decreased in the embryo interactome. Our follow-up on two RNA regulations proteins present in both interactomes, Tral and Cup, revealed that they colocalize with Me31B in nuage granules, P-bodies/sponge bodies, and possibly in germ plasm granules. We further show that Tral and Cup are both needed for maintaining Me31B protein level and mRNA stability, with Tral’s effect being more specific. In addition, we provide evidence that Me31B likely colocalizes and interacts with germ plasm marker Vas in the ovaries and early embryo germ granules. Finally, we show that Me31B’s localization in germ plasm is likely independent of the Osk-Vas-Tud-Aub germ plasm assembly pathway although its proper enrichment in the germ plasm may still rely on certain conserved germ plasm proteins.

## Introduction

Germ cells are essential for sexual reproduction and the survival of many species, and species-specific strategies exist to form germ cells^1–5^. *Drosophila melanogaster* uses maternally inherited germ granules to determine germ cell fate. Germ granules are heterogeneous aggregates of ribonucleoprotein (RNP) complexes^6^ that undergo dynamic positional, morphological, and compositional changes during germline development, a process that spans oogenesis and early embryogenesis^7–11^.

Me31B, a conserved germ granule component^9,12^, is expressed in nurse cells, oocytes, and early embryos^13^. In these cells, Me31B exists in different types of RNP granules, including nuage granules, P-bodies, sponge bodies, and germ plasm granules^12–15^. In these granules, Me31B has been suggested to function as a putative ATP-dependent RNA helicase that interacts with other germline proteins and RNAs to exert post-transcriptional regulation on those RNAs^10,11,13,16,17^. As an important example, Me31B associates with *osk* mRNA to ensure its proper translation into Osk protein only at the posterior pole of developing oocytes. Then, the Osk protein initiates a step-wise assembly pathway that recruits downstream proteins including Vas, Tud, and Aub to form the germ plasm and eventually dictates germ cell formation^13,18–21^.

Me31B exhibits changes in its localization pattern, aggregation status, and even function as germline cells develop during the ovary-to-embryo transition^13,17^. It is believed that these changes are correlated with the different biological contexts in which Me31B exists^17^. Therefore, to understand the role of Me31B during germ cell development, it is important to determine what molecules Me31B interacts with in the germline cells and track how these interactions dynamically change as the cells go through different developmental stages. However, whether and how the Me31B interactome changes from ovaries to early embryos has not been investigated.

In this study, we characterized the Me31B interactome from 0–1 hour embryos and compared it to the previously determined ovary interactome^14^. We found that the Me31B embryo interactome contains RNA regulation proteins including Tral and Cup, glycolytic enzymes, and cytoskeleton/motor proteins like that in the ovaries but contained significantly reduced core germ plasm proteins Vas, Tud, and Aub. The two RNA regulation proteins, Tral and Cup, were found to colocalize with Me31B in different types of RNP granules or show similar localization pattern in the ovaries and early embryos. They were also needed to maintain the Me31B protein level and stabilize *me31B* mRNAs. The reduced Me31B-Vas interaction in the early embryos indicated that Me31B interacts with the germ plasm proteins mainly in the nuage and weakly in the germ plasm. Finally, we showed that germ plasm proteins Osk, Aub, and Dart5 may not be responsible for localizing Me31B to the posterior of an oocyte, but Aub may be still needed for enriching posteriorly localized Me31B in the germ plasm.

## Results and Discussion

### Comparison of the Me31B early embryo interactome and ovary interactome

**Figure 1:**
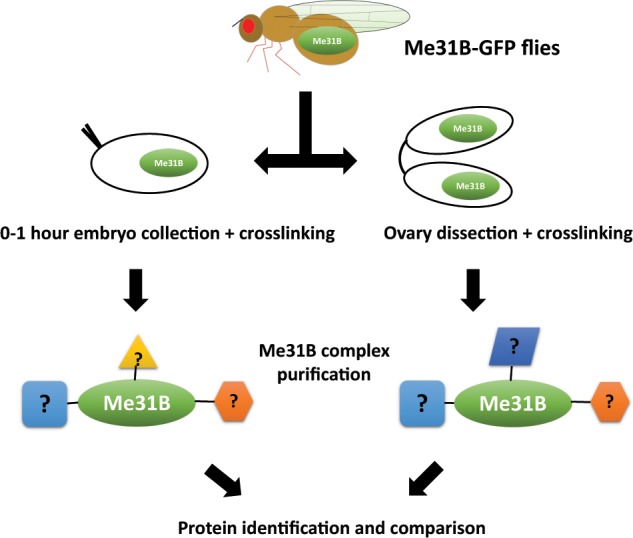
Scheme of the Me31B interactome comparison. Flies expressing Me31B-GFP fusion proteins were used for this study. The 0–1 hour embryos and ovaries were obtained, chemically crosslinked *in vivo* with formaldehyde to stabilize Me31B-GFP proteins to their interacting proteins in the tissues. The crosslinked Me31B-GFP complexes were then immunoprecipitated, gel purified, and subjected to protein identification analysis by mass spectrometry. The embryo interactome and ovary interactome were compared to show the changes of the Me31B interactomes from the two developmental stages. Flies expressing GFP proteins were used as controls (not shown).

To identify the Me31B-interacting proteins in the 0–1 hour embryos, we stabilized Me31B and its interacting partner proteins by *in vivo* chemical crosslinking, isolated the Me31B complexes by immunoprecipitation, and then identified the proteins in the complexes by mass spectrometry (see Materials and Methods and the previous study^22^). The obtained embryo interactome was then compared to the previously determined ovary interactome^14^ to reveal the dynamic changes the Me31B interactome goes through between these two developmental stages (see illustration in Fig. 1 and Materials and Methods). To ensure that a comparable amount of Me31B complexes were used from the two tissues, we examined the Me31B expression in different amounts of crosslinked embryos (50 μl to 400 μl) and used 200 μl of embryos, which yielded a comparable amount of Me31B complexes (Supplementary Fig. 1) as the previous ovary interactome study^14^. To minimize non-specific crosslinking and ensure the specificity and validity of the results, we used 0.2% formaldehyde, which was the lowest concentration of crosslinking reagent that preserved Me31B complexes during stringent IP wash conditions (see Materials and Methods). Also, we conducted four independent biological replicates, and only those protein candidates detected in at least 3 out of the 4 replicates and enriched by more than 2 fold over the control IPs (Supplementary Table 1) were selected. The interactome comparison is summarized in Table 1.

**Table 1:** Comparison between Me31B early embryo interactome and ovary interactome.

Proteins	Embryo	Ovary*	Granule type
***RNA regulation proteins***			
Cup (Cup)	•	•	P body, nuage, germ plasm
Trailer hitch (Tral)	•	•	P body, nuage, germ plasm
Belle (Bel)	•	•	Nuage, germ plasm
Bicaudal-C (BicC)	•	•	P body
Edc3 (Edc3)		•	P body
Pacman (Pcm)	•	•	P body
NOT1 (Not1)		•	P body
eIF4G (eIF-4G)		•	
eIF4E (eIF-4E)	•	•	
***Cytoskeleton and motor proteins***			
Dynein heavy chain (Dhc 64 C)	•	•	Germ plasm
Kinesin heavy chain (Khc)	•	•	
Kinesin light chain (Klc)		•	
β-Tubulin (BetaTub56D)	•	•	
***Glycolytic enzymes***			
Pyruvate kinase (PyK)	•	•	Germ plasm
Phosphoglycerate kinase (PgK)	•	•	Germ plasm
Enolase (Eno)	•	•	
6-phosphofructokinase (PfK)	•	•	
***Germ plasm proteins***			
Tudor (Tud)		•	Nuage, germ plasm
Vasa (Vas)		•	Nuage, germ plasm
Aubergine (Aub)**		•**	Nuage, germ plasm
eIF4A (eIF-4a)	•	•	Germ plasm

“•”Indicates the protein was detected in at least 3 out of the 4 independent repeats and are enriched more than 2 fold over the control. See full data in Supplementary Table 1.

*The ovary Me31B interacting protein data were published previously.

**Aub was reported as a component of Me31B ovary interactome in the previously study, and Aub was detected in 2 out of the 4 independent biological repeats in that study.

We observed that the two interactomes share similarities in three groups of proteins: RNA regulation proteins, glycolytic enzymes, and cytoskeleton/motor proteins. Particularly, we noticed that two RNA-regulation proteins, Tral and Cup, were present in both interactomes and the most abundant proteins in the embryo interactome (Supplementary Table 1). This suggests the presence of Me31B-Tral-Cup complexes throughout oogenesis and early embryogenesis, consistent with previous studies that Me31B, Tral, and Cup form complexes and play vital roles in RNA translational regulation or decay in both the ovaries and early embryos^15,23–28^.

The major difference between the two interactomes is the surprising absence of conserved germ plasm proteins Vas, Tud, and Aub from the embryo interactome (Table 1). A close examination of these proteins’ mass spectrometry data showed that each of them was detected in only 1 of the 4 biological repeats with an extremely low amount (Supplementary Table 1) and our filtering criteria excluded them from the embryo interactome list. Therefore, we interpret that the three proteins’ interactions with Me31B were substantially reduced in the early embryos. This may be explained by the fact that Vas, Tud, and Aub can interact with Me31B in both the nurse cell nuage and oocyte germ plasm in the ovaries^14^, and the nuage structures are apparently missing in the early embryos. This suggests that the Me31B-germ plasm protein interactions occur mostly in the nuage and to a much less extent in the germ plasm.

### Tral and Cup show a similar localization pattern or colocalize with Me31B in ovaries and early embryos

**Figure 2:**
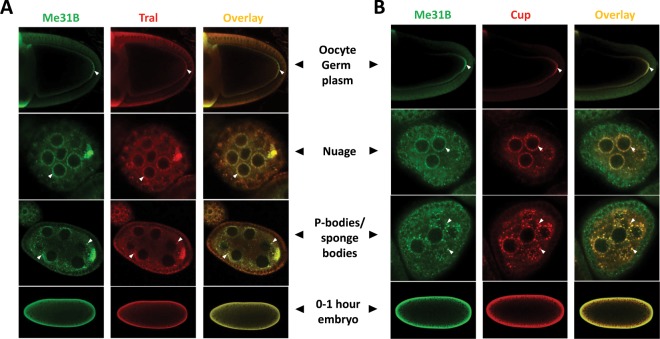
Me31B-Tral and Me31B-Cup show a similar localization pattern or colocalize in the ovaries and early embryos. (**A**) Me31B (green channel) and Tral (red channel) localize similarly in the germ plasm area in stage 9–10 oocytes (arrowheads, top panel). They colocalize in nuage granules (arrowheads, second top panel), P-body granules or sponge bodies (arrowheads, second bottom panel) of nurse cells. Both proteins become dispersed in the 0–1 hour embryo cytoplasm (bottom panel). (**B**) Me31B (green channel) and Cup (red channel) show a similar localization pattern as Me31B and Tral. The oocytes and embryos are positioned dorsal up and posterior right.

The interactome data indicate the presence of Me31B-Tral-Cup complexes in both ovaries and early embryos. Considering that Me31B takes part in several types of germ granules, we wonder whether the Me31B-Tral-Cup complexes also constitute germ granules or other RNPs in the two tissues. To examine this, we performed Me31B-Tral and Me31B-Cup co-immunostaining in the ovaries and 0–1 hour embryos of wild type OR flies. The Me31B-Tral co-staining showed that the two proteins colocalize extensively in the nurse cell nuage granules and nurse cell cytoplasmic granules that appear to be P-bodies or sponge bodies^13,29,30^. Both proteins also showed similar enrichment pattern at the posterior of mid-stage oocyte. Due to the limit of the image resolution, we suggest that their colocalization in the germ plasm granules is possible. Finally, both proteins became dispersed in the cytoplasm of early embryos with extensive overlapping along the cortex (Fig. 2A). For Me31B and Cup, the two proteins showed similar localization relationships in the above structures (Fig. 2B). We conclude that, during oogenesis, Me31B, Tral, and Cup colocalize in the nuage granules, P-body/sponge body granules, and possibly in the oocyte germ plasm granules. Whether the three proteins colocalize in the early embryos cannot be determined because they no longer aggregate into granules and become dispersed throughout the cytoplasm.

### Me31B’s aggregation into the germ granules and Me31B protein level depend on Tral and Cup

**Figure 3:**
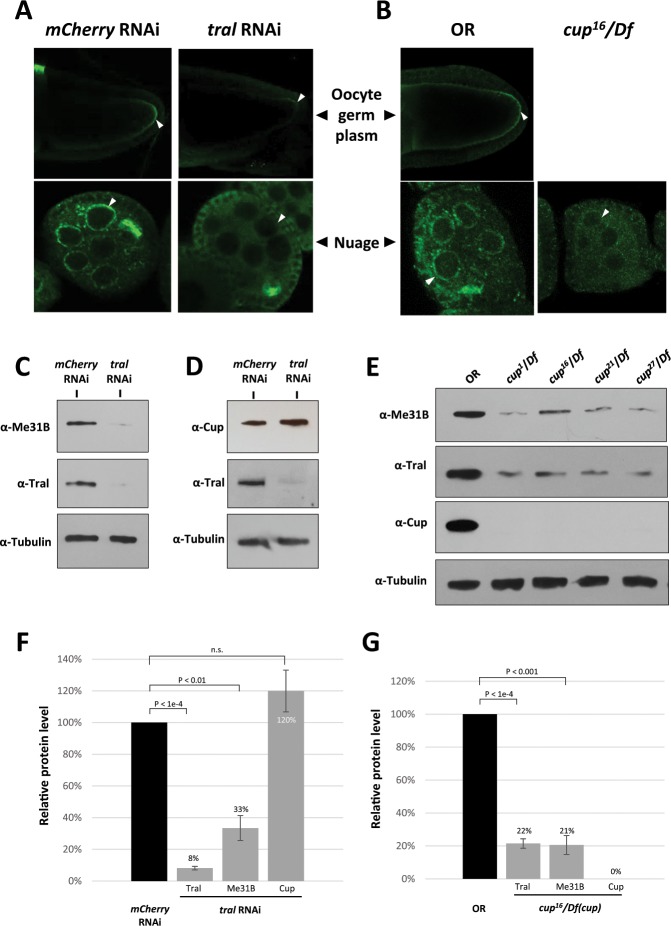
Me31B aggregation into germ granules and Me31B protein levels are decreased in *tral* RNAi and *cup* mutant ovaries. (**A**) Me31B (green channel) localization to oocyte germ plasm granules (arrowheads, top panels) and nuage granules (arrowheads, bottom panels) were substantially reduced in *tral* RNAi egg chambers (right panels) when compared to *mCherry* RNAi control (left panels). **(B)** Me31B protein (green channel) showed lower signal intensity and weaker localization to the nuage of *cup*^16^/*Df* egg chambers (arrowheads, right panel) when compared to OR wild type (arrowheads, left panels). Note that *cup*^16^/*Df* egg chambers arrest at approximately stage 5, so late-stage oocytes were not observed. **(C)** *tral* RNAi ovaries (right lane) showed reduced Me31B protein level and Tral protein level when compared to *mCherry* RNAi controls (left lane). **(D)** *tral* RNAi ovaries (right lane) do not show a decrease of Cup protein when compared to *mCherry* RNAi control (left lane). **(E)** *cup*^1^*/Df*, *cup*^16^*/Df*, *cup*^21^*/Df*, and *cup*^27^*/Df* ovaries (right lanes) showed decreased Me31B and Tral protein levels compared to OR (left lane). **(F)** Quantification of relative Tral, Me31B, and Cup protein levels in *tral* RNAi ovaries comparing to *mCherry* RNAi controls. **(G)** Quantification of relative Tral, Me31B, and Cup protein levels in *cup*^16^/*Df* ovaries comparing to OR controls. For the Western blots, the images are cropped to show only the relevant protein bands, and the full-size blots are presented in Supplementary Fig. 2. The images shown are representative images of multiple biological replicates, and the additional biological replicate images are presented in Supplementary Fig. 3. Western blot quantifications were normalized by using the alpha-tubulin proteins as loading controls. n.s., not statistically significant.

Next, we asked whether Me31B expression or localization to the germ granules is affected by the absence of Tral or Cup. Due to the difficulty of obtaining sufficient *tral* mutant flies, we used *tral* RNAi strain +MTD-GAL4 (Maternal Triple Driver) strain to generate germline Tral-knockdown flies. The *tral* RNAi flies showed an effective Tral knockdown (−92%, *p* < 0.0001) in the ovaries (Fig. 3C,F) and their eggshells displayed similar dorsal-ventral patterning defects (Supplementary Table 2) as previously reported strong *tral* mutants^31^. Anti-Me31B immunostaining in the *tral* RNAi ovaries showed conspicuously weaker Me31B localization to the nuage granules, P-bodies/sponge bodies, and the oocyte germ plasm granules when compared to the *mCherry* RNAi control (Fig. 3A). We further confirmed a decrease of Me31B protein level (−67%, *p* < 0.01) in the *tral* RNAi fly ovaries by Western blot (Fig. 3C,F). We reason that the observed Me31B localization defects in the *tral* RNAi flies could be caused by the decreased Me31B protein abundance. Interestingly, however, Tral knockdown did not cause a decrease of Cup (+20%, *p* > 0.1, not statistically significant, Fig. 3D,F), suggesting that Tral affects the Me31B protein level specifically.

We went on to analyze whether the absence of Cup influences Me31B expression and localization. We performed anti-Me31B immunostaining in the ovaries from *cup*^16^/*Df* flies, a protein-null *cup* mutant^26^. We noticed reduced Me31B signal intensity and weaker localization to the nuage granules when compared to wild type (Fig. 3B). Me31B localization to oocyte germ plasm granules in the mutant could not be determined because of its early-stage (~ stage 5) egg chamber degeneration. We confirmed the Me31B protein level decrease in the *cup*^16^/*Df* mutant by Western blot (−79%, *p* < 0.001, Fig. 3E,G). Similar phenotypes were observed in three other *cup* mutants, *cup*^1^*/Df*, *cup*^21^*/Df*, and *cup*^27^*/Df* (data not shown and Fig. 3E). Interestingly, like Me31B, Tral protein was also reduced in the *cup*^16^/*Df* mutant (−79%, *p* < 0.0001, Fig. 3E,G).

We conclude that Me31B protein level is under the regulation of both Tral and Cup, and these two proteins seem to render their effects differently. Tral knockdown substantially decreased the Me31B protein level without affecting Cup (Fig. 3C,D), suggesting Tral’s specific role in maintaining the Me31B level. However, *cup* mutations reduced Tral and Me31B protein level to a similar extent (Fig. 3E,G), suggesting that Cup’s regulation on the two proteins may be indiscriminate. Also, we do note the possibility that Cup may have no direct influence on Me31B, and *cup* mutation decreased Me31B protein level indirectly through Tral.

Furthermore, we tentatively conclude that the Me31B germ granule localization/aggregation defects in the Tral-knockdown and *cup* mutant flies (Fig. 3A,B) were likely caused by the decreased Me31B protein levels, which may be simply explained by the general principle of binding reactions. This is consistent with the observation that Me31B proteins exist as granular aggregates in the ovaries but then become dispersed in the early embryos (Fig. 2 and previous study^13^), in accordance with earlier reports that Me31B protein level decreases rapidly in the early embryos and at the maternal-to-zygotic transition (MTZ)^17,32^. Alternatively, previous studies reported that certain germ granule RNA-binding proteins can undergo a concentration- and RNA-dependent phase transition from a soluble to viscous state^20,33–37^, and Me31B, a putative RNA-helicase, may use a similar concentration-dependent mechanism to aggregate into structures like the germ granule RNPs.

### Cup and Tral are needed for the stability of *me31B* mRNA

**Figure 4:**
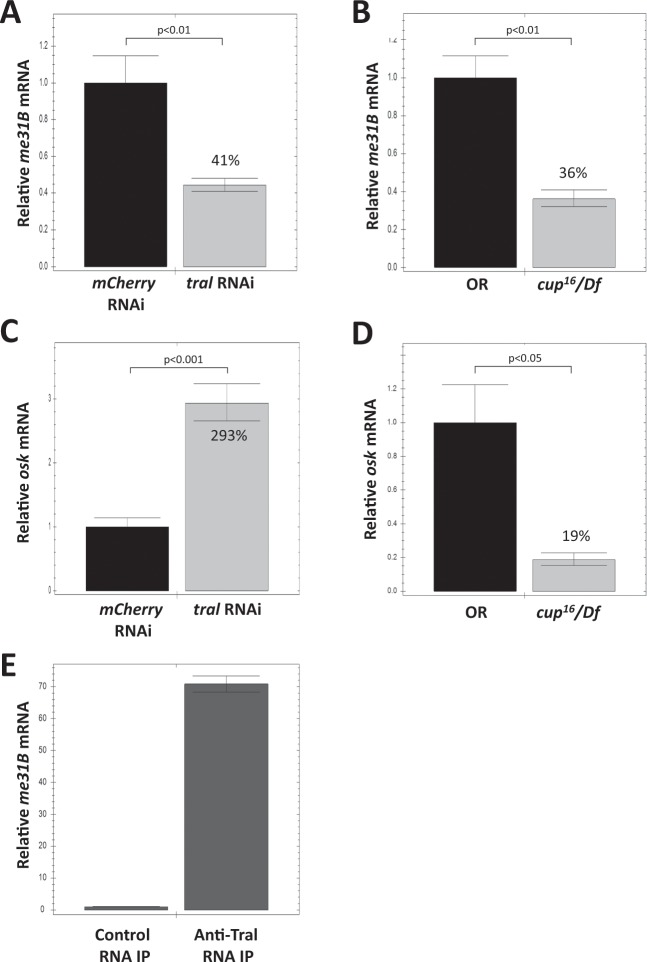
Quantitative RT-PCR analysis of *me31B* mRNA and *osk* mRNA in *tral* RNAi ovaries, *cup* mutant ovaries, and anti-Tral IP. (**A**) *me31B* mRNA showed a 59% decrease in *tral* RNAi ovaries when compared to *mcherry* RNAi control. **(B)** *me31B* mRNA showed a 64% decrease in *cup*^16^/*Df* mutant when compared to OR control. **(C)** *osk* mRNA showed a 293% increase in *tral* RNAi ovaries when compared to *mcherry* RNAi control. **(D)** *osk* mRNA showed a 81% decrease in *cup*^16^/*Df* mutant when compared to OR control. Error bar represents the standard error of the mean. The percentage numbers on the bars represent their relative values to the controls. **(E)** RNA IP with anti-Tral antibodies contained 70-fold more *me31B* mRNA than the control IP. Error bar represents standard deviation.

Considering Tral and Cup’s role in RNA post-transcriptional regulation^24,26,31,38,39^, we hypothesize that they could have influenced the Me31B expression by regulating the stability of *me31B* mRNA. To test this, we examined the *me31B* mRNA level by quantitative RT-PCR in the ovaries of the *tral* RNAi and the *cup*^16^/*Df* mutant flies. Indeed, we observed a significant *me31B* mRNA decrease in the *tral* RNAi flies (−59%, *p* < 0.01, Fig. 4A) and a slightly greater decrease in the *cup*^16^/*Df* mutant (−63%, *p* < 0.01, Fig. 4B) when compared to their controls, respectively. We conclude that Tral and Cup are both needed for the stability of *me31B* mRNAs in the ovaries. We do note that part of the *me31B* mRNA decrease in the *cup* mutant may come from the indirect contribution of Tral protein decrease.

Tral’s regulation on *me31B* mRNA raised the possibility that Tral protein physically interacts with the RNA. To test this, we performed RNA immunoprecipitation (RIP) from wild type ovaries with anti-Tral antibodies and examined the *me31B* mRNA in the precipitates by qRT-PCR. As expected, the anti-Tral precipitate showed a 70-fold enrichment of *me31B* mRNA over the control IP (Fig. 4E), suggesting a direct interaction between Tral and *me31B* mRNA.

Since Me31B, Tral, and Cup form RNA regulation complexes and Cup is known to stabilize the important germline mRNA *osk* during oogenesis^26^, we asked whether Me31B and Tral also play a part in stabilizing *osk* mRNA. To this end, we examined the *osk* mRNA level in the ovaries of the *tral* RNAi flies. Surprisingly, we observed a significant *osk* mRNA level increase when compared to control (+293%, *p* < 0.001, Fig. 4C). Considering that *tral* RNAi causes a significant decrease of Tral proteins (−92%) as well as Me31B (−67%) (Fig. 3C,F), we argue that Tral and its downstream protein Me31B are not needed for *osk* mRNA stability. In contrast, *cup*^16^/*Df* mutant showed a significant and strong decrease of *osk* mRNA (−81%, *p* < 0.05, Fig. 4D and similar result in a previous study^26^). We conclude that, unlike Cup, Tral and Me31B are not essential for stabilizing *osk* mRNA, which also suggests that Tral’s RNA-stabilizing effect towards *me31B* mRNA is specific.

### Me31B likely colocalizes with Vas in the ovary and early embryo germ granules

**Figure 5:**
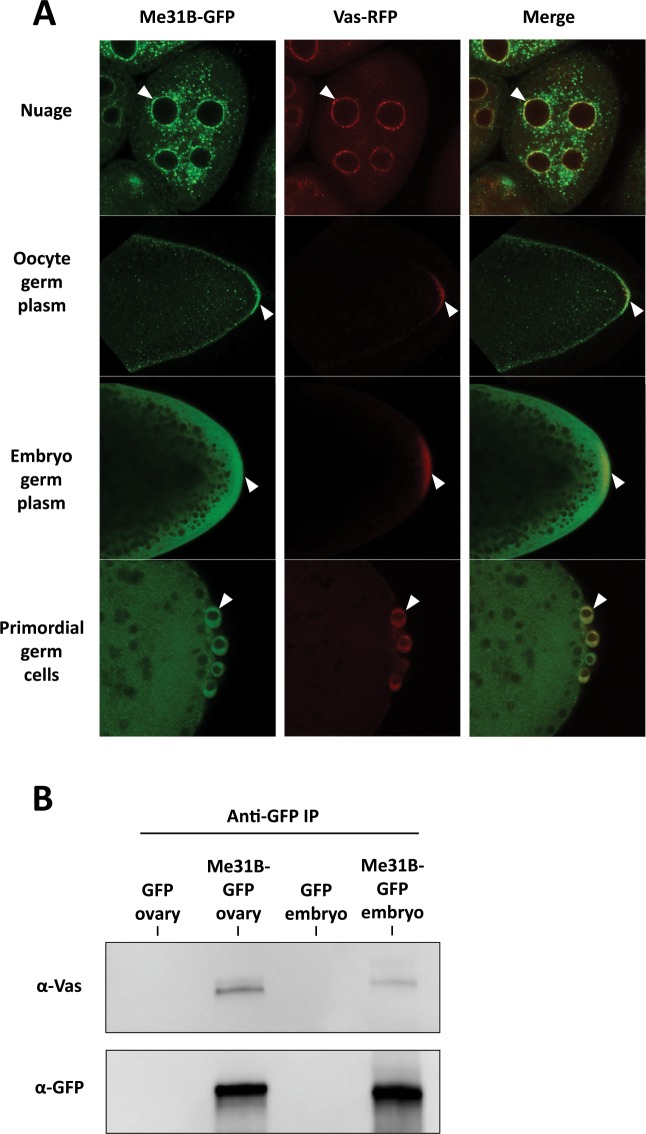
Me31B-Vas localization and interactions in the ovaries and early embryos. (**A**) In *me31B-GFP* and *vas-RFP* double endogenously tagged fly ovaries and embryos, Me31B-GFP likely colocalizes with Vas-RFP in the nuage granules (arrowheads, top panels) and oocyte germ plasm granules (arrowheads, second top panels). Me31B showed a dispersed distribution in the embryo germ plasm (arrowheads, second bottom panels) and primordial germ cells (arrowheads, bottom panels), and its colocalization with Vas-RFP granules appear not as obvious as in the ovaries. A higher magnification image of embryo germ plasm is presented in Supplementary Fig. 4 to better illustrate the Me31B-GFP and Vas-RFP granules. **(B)** Anti-GFP immunoprecipitation pulled down Me31B-GFP and co-immunoprecipitated Vas from the *me31B-GFP* fly ovaries and embryos. The Vas coimmunoprecipitated from the embryos (last lane) was lower than that from the ovaries (second lane). GFP-expressing fly tissues were used as controls (first and third lanes). For the Western blots, the images are cropped to show only the relevant protein bands, and the full-size blots are presented in Supplementary Fig. 5.

The fact that core germ plasm proteins, Vas, Tud, and Aub, were barely detectable in the Me31B embryo interactome raised a suspicion about their association with Me31B in the embryo germ plasm. Although our previous study demonstrated that Me31B has a similar localization pattern as Vas, Tud, and Aub in the nuage and germ plasm in the ovaries^14^, whether this is true in the early embryos is not clear. To this end, we generated flies with double endogenous tags, Me31B-GFP and Vas-RFP (see Materials and Methods), to analyze the localization relationships between native Me31B and Vas. Vas was used here as a reliable germ granule marker like many previous studies^8,20,40,41^. In the ovaries of the double-tagged flies, Me31B-GFP proteins aggregated into granular structures in the nuage of nurse cells and oocytes. These granules accumulated along the oocyte cortex and became obviously enriched in the germ plasm where they seemed to colocalize with Vas-RFP granules (Fig. 5A). However, in the early embryos and primordial germ cells, Me31B-GFP appeared dispersed rather than aggregated, and its enrichment in the germ plasm and colocalization pattern with Vas seem less obvious than that in the oocyte (Fig. 5, compare top two panels with bottom two panels and Supplementary Fig. 4). This observation may be caused by Me31B being dispersed in the embryo cytoplasm and due to the limits of the fluorescence microscope. Since a previous study using immuno-electron microscopy provided convincing evidence that Me31B is 7-fold concentrated in embryo germ plasm^12^, we believe that the Me31B-GFP proteins, or a portion of the proteins in the embryo germ plasm area, still likely colocalize with the Vas-RFP granules. Further support of Me31B-Vas interaction in the germ plasm comes from the finding that Me31B and Vas can be co-immunoprecipitated from early embryos (Fig. 5B). We note that the Vas co-immunoprecipitated with Me31B-GFP from the embryos seemed less than that from the ovaries (Fig. 5B), consistent with the reduced Me31B-Vas interaction in the embryos indicated by the interactome analysis (Table 1 and Supplementary Table 1). This also suggests that Me31B and the germ plasm proteins interact mainly in the nuage and only weakly in the germ plasm. Collectively, we conclude that Me31B likely colocalizes and interacts with Vas in the nuage, oocyte germ plasm granules, and early embryo germ plasm.

### Me31B localization into germ granules is independent of Osk and Vas, but its proper enrichment in the germ plasm may still rely on the conserved germ plasm proteins

**Figure 6:**
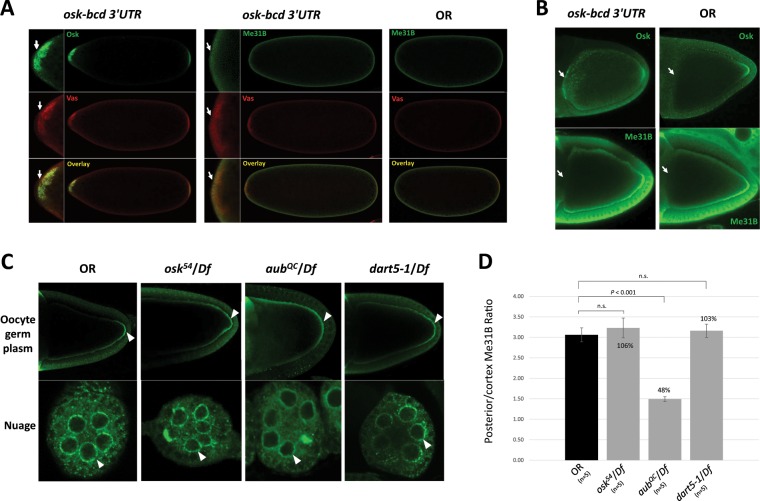
Me31B localization is not affected in *osk-bcd 3*′*UTR* embryos and ovaries and *osk*, *aub*, *dart5* mutants, but its enrichment level in the posterior of the *aub* mutant oocytes is affected. (**A**) Anteriorly expressed Osk in *osk-bcd 3*′*UTR* embryos recruited Vas to the anterior (arrows, left panels) but did not induce obvious accumulation of Me31B to the anterior (arrows, mid panels). Me31B exhibited similarly uniform distribution in both *osk-bcd 3*′*UTR* (mid panels) and OR embryos (right panels). Mini pictures represent the magnified anterior region of the embryos. **(B)** Anterior accumulation of Osk was detected in *osk-bcd 3*′*UTR* but not in OR oocytes (arrows, top panels). However, no anterior accumulation of Me31B was detected in *osk-bcd 3*′*UTR* nor OR oocytes (arrows, bottom panels). The two anti-Me31B images were slightly over-exposed to help detect potential anterior Me31B signals. **(C)** Me31B localization to the posterior and cortex of oocytes (arrowheads, top panels) and nuage granules (arrowheads, bottom panels) in *osk*^54^/*Df*, *aub*^*QC*^/*Df*, and *dart5-1*/*Df* ovaries are similar to the OR control. However, the Me31B enrichment levels at the posterior over that along the cortex in *aub*^*QC*^*/Df* is significantly lower than the control. **(D)** The Me31B posterior enrichment levels (see Materials and Methods) in the *osk*^54^/*Df*, *aub*^*QC*^/*Df*, *dart5-1*/*Df* mutants are 106%, 48%, and 103% relative to the control, respectively. Error bar represents the standard error of the mean.

Considering Vas’s and its upstream protein Osk’s essential roles in germ plasm assembly^18,20,42^, we next asked whether they are responsible for recruiting Me31B into the germ plasm granules. To answer this, we took advantage of the *osk-bcd3*′*UTR* transgenic strain which expresses Osk ectopically at the anterior of the embryo. The anterior Osk then recruits downstream proteins including Vas, Tud, and Aub and induces the assembly of germ plasm there^18,19^. We hypothesized that Me31B accumulation would occur in the anteriorly assembled germ plasm of *osk-bcd3*′*UTR* embryos should Osk or Vas recruit Me31B. Surprisingly, the anterior Osk induced the accumulation of Vas in the transgenic embryos but did not induce obvious accumulation of Me31B (Fig. 6A). Instead, Me31B showed a uniform distribution in the *osk-bcd3*′*UTR* embryos like that in the wild type (Fig. 6A). In the *osk-bcd3*′*UTR* oocytes, similar results were observed: despite the anterior expression of Osk, no obvious anterior localization or accumulation of Me31B was detected, and Me31B was distributed along the cortex of the oocytes with enrichment at the posterior like that in the wild type (Fig. 6B). This suggests that Me31B’s localization into the germ plasm could be independent of Osk and Vas, which is consistent with our previous finding that Me31B’s localization to nuage and germ plasm was unaffected in *vas* mutant flies^14^.

To lend further support, we analyzed Me31B’s localization pattern in *osk* mutant (*osk*^54^/*Df(3R)p*^*XT103*^), *aub* mutant (*aub*^*QC*^/*Df(2L)BSC145*), and *dart5* mutant (*dart5-1*/*Df(2R)Jp7*) fly ovaries. *osk*^54^ allele does not produce Osk protein at the posterior, so germ plasm does not form in the *osk*^54^/*Df(3R)p*^*XT103*^ flies^43^. *aub*^*QC*^ is a strong loss-of-function allele of *aub* gene^44^. The *dart5* gene encodes a methyl-transferase that methylates Aub to allow the binding of Tud^45–47^ during germ granule assembly and is therefore also analyzed here. The *dart5-1* allele does not produce any full-length protein^48^. In the *osk*, *aub*, and *dart5* mutants, Me31B localized similarly as the wild type, to the nuage of nurse cells, along the oocyte cortex, and at the posterior of the oocytes (Fig. 6C). Note that, in the *aub*^*QC*^/*Df* mutant oocyte, the posterior and cortex Me31B appear more diffused (Fig. 6C). To examine whether Me31B posterior enrichment level is affected in the mutants, we measured the fluorescence intensity ratio of the posterior Me31B over that along the cortex (see Materials and Methods) and used it as an estimation of Me31B enrichment level in the posterior germ plasm. We do note that these measurements are only estimation and are subjected to technical limitations like the quality of sample staining and the selection of the microscope focal planes. We found that the *aub* mutant showed a 52% lower Me31B posterior enrichment level than the wild type (*p* < 0.01), while the *osk* and *dart5-1* mutants showed minor and non-significant changes (Fig. 6D). To summarize, although Me31B’s localization to the posterior of an oocyte is likely independent of Osk, Aub, and Dart5, its proper enrichment at the site may still rely on Aub. Together with our previous report that Me31B’s localization pattern is not affected in *vas* and *tud* mutants^14^, we speculate that Me31B’s localization in a developing oocyte may be independent of the Osk-Vas-Tud-Aub assembly pathway, but its proper enrichment at the posterior germ plasm may still depend on certain conserved germ plasm proteins like Aub.

**Figure 7:**
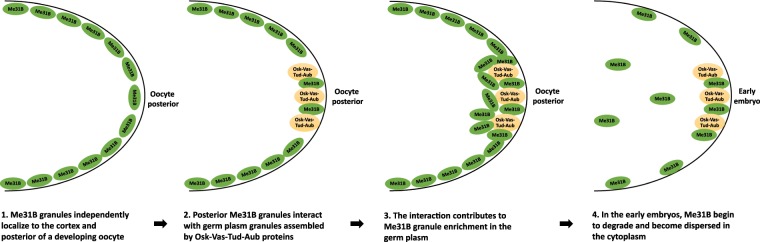
Hypothetical model of Me31B localization and enrichment into germ plasm. In this model, (1) Me31B (complexed with Tral and Cup, not shown) transport and localize along the cortex and to the posterior of a developing oocyte. (2) The posteriorly localized Me31B proteins exist in distinct granules from the Osk-Vas-Tud-Aub marked germ plasm granules, but they exist in close proximity and interact in the germ plasm. Step 1 and 2 can occur at the same time and do not necessarily happen in the shown order. The two steps are separated here to illustrate Me31B’s independent localization mechanism. (3) The Me31B granule-germ plasm granule interaction is needed for Me31B granules to accumulate/enrich in the germ plasm. (4) In the early embryo, Me31B proteins begin to degrade rapidly, lose aggregation status, and become dispersed in the cytoplasm.

This speculation, together with earlier conclusions in this study, led us to propose a hypothetical model for Me31B localization and enrichment process in the germline cells (Fig. 7). In this model, Me31B and conserved germ plasm proteins, Osk-Vas-Tud-Aub, exist in distinct granules in the germ plasm, Osk-Vas-Tud-Aub in germ plasm granules and Me31B (possibly associated with Tral and Cup) in separate granules but in close proximity. Me31B granules use an Osk-Vas-Tud-Aub-independent mechanism to localize to the cortex and the posterior of a developing oocyte, then the posteriorly localized Me31B granules interact with the germ plasm granules, which is necessary for proper Me31B granule enrichment in the germ plasm. In the early embryos, Me31B proteins begin to degrade rapidly^17^ and become dispersed in the cytoplasm.

## Materials and Methods

### Drosophila strains

*me31B-GFP* (stock 51530), *aub*^*QC*^ (stock 4968), *Df(2L)BSC145* (stock 9505), *Df(2L)BSC7* (stock 6374), *Df(2R)Jp7* (stock 3522)*, mCherry* RNAi (stock 35787), *tral* RNAi (stock 38908), MTD-GAL4 (Maternal Triple Driver, stock 31777) strains were obtained from Bloomington Drosophila Stock Center. The *dart5-1* strain was previously described^48^. The *vas-RFP*, *osk*^54^, *and Df(3R)p*^*XT103*^ strains were received from Dr. Elizabeth R. Gavis lab and were previously described^49,50^. OR wild type strain and *GFP* strain used as controls were previously described^12^. *osk-bcd 3*′*UTR* strain was received from Dr. Ruth Lehmann lab and was previously described^19^. *cup*^1^, *cup*^16^, *cup*^21^, and *cup*^27^ strains were received from Dr. Akira Nakamura lab.

### *Drosophila* embryo chemical crosslinking and Me31B complex purification

The embryo crosslinking and the purification of the crosslinked Me31B complexes were performed essentially as previously described^14,22^. Briefly, 0–1 hour embryos from Me31B-GFP flies or GFP control flies were dechorionated by 50% bleach, permeabilized by heptane, and then crosslinked with 0.2% formaldehyde. The crosslinked embryos were quenched in 0.25 M glycine, washed in PBS + 0.1% Triton X-100, and then stored at –80 °C for later use. 200 μl crosslinked Me31B-GFP and GFP control embryos were homogenized in lysis buffer, adjusted to equal concentrations, and then incubated with anti-GFP magnetic beads (Chromotek). After 5 stringent washes of the beads, the bound complexes were eluted with SDS-sample buffer at 65 °C for 10 minutes. The complexes were resolved on a 3–15% step gel, and the gel band containing the Me31B complexes, as well as the band in the GFP control, were excised and subjected to protein identification analysis by mass spectrometry.

### Immunohistochemistry

*Drosophila* ovary immunostaining was performed as previously described^14,51,52^. The following antibody dilutions were used: rabbit-anti-Me31B (1:2,200), rat-anti-Vas (Developmental Studies Hybridoma Bank) (1:100), mouse-anti-Cup (1:1,000), rat-anti-Tral (1:1,000), and rabbit-anti-Osk (1:2,000). Goat-anti-rabbit-Alexa 488 secondary antibody (Jackson ImmunoResearch) and donkey-anti-rat-Cy3 secondary antibodies (Jackson ImmunoResearch) were both used at 1:500. Images were captured by an Olympus FV3000 confocal laser scanning microscope.

For *Drosophila* embryo immunostaining, the procedure was similar to the ovary immunostaining described above except for the embryo treatment procedures as follows. 0–1 hour embryos (~20 μl) were collected and incubated in 50% bleach solution for 3 minutes. The embryos were then rinsed with water to remove the bleach and then rinsed with PBS + 0.1% Tween for 2 minutes. The embryos were rinsed two more times with water and then fixed in Fixative (equal volume of 4% formaldehyde in PBS + 0.05% Tween and Heptane) on a rotary shaker for 25 minutes. After replacing the Fixative with an equal amount of methanol, the embryos were immediately shaken vigorously for 15 seconds to remove the vitelline membrane. The embryos were rinsed with methanol three more times and then stored at −20 °C in methanol until use. Before antibody staining, the embryos were rehydrated by washing with PBS + 0.2% Tween for 3 times, 5 minutes each. The rest of the antibody staining procedure was the same as the ovary immunostaining. The treatment for Me31B-GFP/Vas-RFP double-tagged embryos was slightly different as follows. After removing the vitelline membrane, the embryos were immediately rehydrated by incubating in 75%, 50%, 25%, 0% methanol in PBS + 0.2% Tween. Then, the embryos were mounted onto slides in mounting medium.

### Immunofluorescence image analysis

Fluorescence images were analyzed by using ImageJ (https://imagej.nih.gov/ij/). The length of the posterior germ plasm-localized Osk protein signals in the OR oocyte was measured and divided by the total width of the oocyte (Fig. 6B, anti-Osk). The obtained ratio, 10.7% (from the posterior end of the oocyte), was used as the posterior/cortex division point of the anti-Me31B-stained OR, *osk*^54^/*Df*, *aub*^*QC*^/*Df*, and *dart5-1*/*Df* oocytes. The Me31B posterior enrichment level was calculated by dividing the mean Me31B signal intensity of the posterior portion with that of the cortex portion. The average intensity of the lateral and dorsal cortex was used for the ratio calculation. In the case one side of the cortex was apparently under-stained, only the other side of the cortex was used for the ratio calculation.

### Coimmunoprecipitation

For Me31B-GFP and Vas co-IP experiment, non-crosslinked ovaries and embryos were used. 200 μL dechorionated Me31B-GFP and GFP embryos as well as 20 μL Me31B-GFP and GFP ovaries were homogenized in lysis buffer (10 mM Tris/Cl pH 7.5, 250 mM NaCl, 0.5 mM EDTA, 0.5% NP-40, and protease inhibitors). After removing cellular debris by centrifugation, the protein concentrations of the lysates were determined by using Bradford Reagent (BioRad), and the lysates were diluted to 2 mg/ml (embryo lysates) and 1 mg/ml (ovary lysates), respectively. Then, each lysate was incubated with 10 μL anti-GFP magnetic beads (Chromotek) with agitation for 1 hour at room temperature. After 3 washes with stringent wash buffer (10 mM Tris/Cl pH 7.5, 250 mM NaCl, 0.5 mM EDTA) to the beads, the bound protein complexes were eluted with SDS-sample buffer at 95 °C for 5 minutes. The eluted proteins were then analyzed by Western blots.

### Western blots

Western blot antibodies were used at the following dilutions: rabbit-anti-Me31B (1:20,000), mouse-anti-Cup (1:2,000), rabbit-anti-Tral (1:2,000), rat-anti-Tral (1:4,000), rabbit-anti-GFP (1:100,000), rat-anti-Vas (1:2,000), and mouse-anti-α-Tubulin (1:100,000). Secondary antibodies were used at the following dilutions: mouse-anti-rabbit HRP (Jackson ImmunoResearch) (1:10,000 for rabbit-anti-Me31B primary antibody, 1:5,000 for rabbit-anti-Tral, 1:10,000 for rabbit-anti-GFP), goat-anti-mouse HRP (Santa Cruz Biotechnology) (1: 50,000 for mouse-anti-α-Tubulin primary antibody, 1:5,000 for mouse-anti-Cup primary antibody), goat-anti-rat HRP (Jackson ImmunoResearch) (1:10,000 for rat-anti-Tral primary antibody, and 1:5,000 for rat-anti-Vas). The protein band quantification analysis was performed by using ImageJ (https://imagej.nih.gov/ij/).

### RNA extraction, cDNA synthesis, and quantitative RT-PCR

Ovarian total RNA was extracted from 5 µl freshly dissected fly ovaries by using Qiagen RNeasy Purification Kit (Qiagen) according to the manufacturer’s instructions. The obtained RNA samples’ concentrations were measured by using NanoDrop 2000c. The RNAs were reversely transcribed to cDNAs by using High Capacity cDNA Reverse Transcription Kit (ThermoFisher) according to the manufacturer’s instructions. The synthesized cDNAs were then used for quantitative PCR by using Luna Universal qPCR Master Mix (New England Biolabs). The following PCR Primers were used in this study: *osk* forward 5′- TTGCTGAGCCACGCCCAGAA-3′, *osk* reverse 5′-ACATTGGGAATGGTCAGCAGGAAATC-3′, *me31B* forward 5′-CTGCCGCCAAAGGACAACC-3′, *me31B* reverse 5′-GCTATAGGAATAGCTGCTTCCTG-3′, *rp49* forward 5′-GCTAAGCTGTCGCACAAA, *rp49* reverse 5′-TCCGGTGGGCAGCATGTG-3′. *rp49* RNA was used as the reference. Data analysis was conducted by using the CFX Manager Software (BioRad) and Microsoft Excel.

### RNA immunoprecipitation

100 µL of OR ovaries were homogenized in Lysis Buffer (150 mM KCl, 0.05% NP-40, 20 mM HEPES-KOH pH 7.4, 1 mM DTT, 1 mM MgCl2, Protease Inhibitor, RNase Inhibitor). Cellular debris was removed through centrifugation. The total protein concentration of the lysate was determined by using a Bradford Assay and adjusted to 1.0 mg/mL. Then the lysate was divided into two aliquots. To one aliquot, 10 µg of rat-anti-Tral antibody were added, while RNase-free water was added to the other aliquot as control. The lysates were then incubated overnight (16 to 24 hours) on an end-over-end shaker at 4 °C. The two lysates were separately incubated with 25 µl Protein G magnetic agarose beads (ThermoFisher) and incubated on an end-over-end rotator at 4 °C for 2 hours. The beads were then collected and washed with Wash Buffer (150 mM KCl, 0.1% NP-40, 20 mM HEPES-KOH pH 7.4, 1 mM DTT, 1 mM MgCl2, Protease Inhibitor, RNase Inhibitor) 4 times, 5 minutes each time, and finally with RNase-free water for one time. RNAs bound to the beads were extracted by using TRIzol (ThermoFisher) according to the manufacturer’s instructions. The extracted RNAs were analyzed by cDNA synthesis and qRT-PCR methods described earlier.

## Supplementary information

Supplementary Information.

## Data Availability

All data generated or analyzed during this study are included in this published article (and its Supplementary Information files).
