# Supplementary material for: Comparative Proteomics Reveal Me31B’s Interactome Dynamics, Expression Regulation, and Assembly Mechanism into Germ Granules during Drosophila Germline Development
Source: Sci Rep. 2020 Jan 17;10:564. doi: 10.1038/s41598-020-57492-y (PMC6969142; doi:10.1038/s41598-020-57492-y)
Supplement: Supplementary file 1 — Supplementary Information. [file 41598_2020_57492_MOESM1_ESM.pdf]

Supplementary Information for Manuscript:

Comparative Proteomics Reveal Me31B's Interactome Dynamics, Expression Regulation, and Assembly Mechanism into Germ Granules during *Drosophila* Germline Development

Aidan McCambridge[1], Dhruv Solanki[1], Nicholas Olchawa[1], Neal Govani[1], Jonathan C. Trinidad[2], and Ming Gao[1,*]

1. Biology Department, Indiana University Northwest, Gary, IN, USA
2. Department of Chemistry, Indiana University, Bloomington, IN, USA

Supplementary Table 1. Proteins identified in embryo Me31B complexes

Proteins	Granule type	Uniprot_ID	Me31B IP #1*	Me31B IP #2	Me31B IP #3	Me31B IP #4	GFP IP #1	GFP IP #2	GFP IP #3	GFP IP #4	Me31B/GFP ratio**	Me31B IP average***	Detected equal to or more than 3 times in Me31B IPs
RNA regulation proteins													
Cup (Cup)	P body, nuage, germ plasm	CUP_DROME	27	6	106	80	0	0	0	2	109.50	54.75	Yes
Trailer hitch (Tral)	P body, nuage, germ plasm	M9PF14_DROME	61	28	152	119	0	0	3	11	25.71	90.00	Yes
Belle (Bel)	Nuage, germ plasm	DDX3_DROME	5	3	24	13	0	0	0	0	High	11.25	Yes
Bicaudal-C (BicC)	P body	BICC_DROME	2	0	3	5	0	0	0	0	High	2.50	Yes
Edc3 (Edc3)	P body	EDC3_DROME	2	2	0	0	0	0	0	0	High	1.00	No
Pacman (Pcm)	P body	Q9VWI1_DROME	4	3	11	8	0	0	0	0	High	6.50	Yes
NOT1 (Not1)	P body	A8DY81_DROME	0	0	17	4	0	0	0	0	High	5.25	No
eIF4G (eIF-4G)		O61380_DROME	9	0	28	0	0	0	2	0	18.50	9.25	No
eIF4E (eIF-4E)		IF4E_DROME	1	1	4	5	0	0	0	0	High	2.75	Yes
Cytoskeleton and motor proteins													
Dynein heavy chain (Dhc 64C)	Germ plasm	DYHC_DROME	3	1	17	16	0	0	0	0	High	9.25	Yes
Kinesin heavy chain (Khc)		KINH_DROME	7	5	11	29	0	0	0	7	7.43	13.00	Yes
Kinesin light chain (Klc)		KLC_DROME	0	0	1	0	0	0	0	0	High	0.25	No
β-Tubulin (BetaTub56D)		TBB1_DROME	34	32	72	79	6	4	22	30	3.50	54.25	Yes
Glycolytic enzymes													
Pyruvate kinase (PyK)	Germ plasm	KPYK_DROME	8	15	41	62	0	3	6	9	7.00	31.50	Yes
Phosphoglycerate kinase (PgK)	Germ plasm	PGK_DROME	2	6	9	16	0	0	0	4	8.25	8.25	Yes
Enolase (Eno)		ENO_DROME	0	4	9	13	0	0	2	10	2.17	6.50	Yes
6-phosphofructokinase (PfK)		PFKA_DROME	0	7	17	22	0	0	1	1	23.00	11.50	Yes
Germ plasm proteins													
Tudor (Tud)	Nuage, germ plasm	TUD_DROME	0	0	0	2	0	0	0	0	High	0.50	No
Vasa (Vas)	Nuage, germ plasm	VASA1_DROME	0	2	0	0	0	0	0	0	High	0.50	No
Aubergine (Aub)	Nuage, germ plasm	AUB_DROME	0	0	0	1	0	0	0	0	High	0.25	No
eIF4A (eIF-4a)	Germ plasm	IF4A_DROME	8	10	24	34	1	0	4	10	5.07	19.00	Yes

*Numbers shown for a given protein's IP correspond to the numbers of t

**Average number of peptides in the Me31B IP divided by the average number of peptides in the GFP control. If no protein was found in the GFP control, this enrichment ratio was labeled as "high".

***Average number of peptides in the Me31B Ips

Supplementary Table 2. *tral* RNAi embryo dorsal appendage number phenotypes

Number of dorsal appendages	*mcherry* RNAi (n=500)	*tral* RNAi (n=503)
2	99.6%	14.1%
1	0.0%	29.2%
0	0.4%	56.7%

Supplementary Figure 1. Me31B complexes isolated from the early embryos were comparable to that from the ovaries

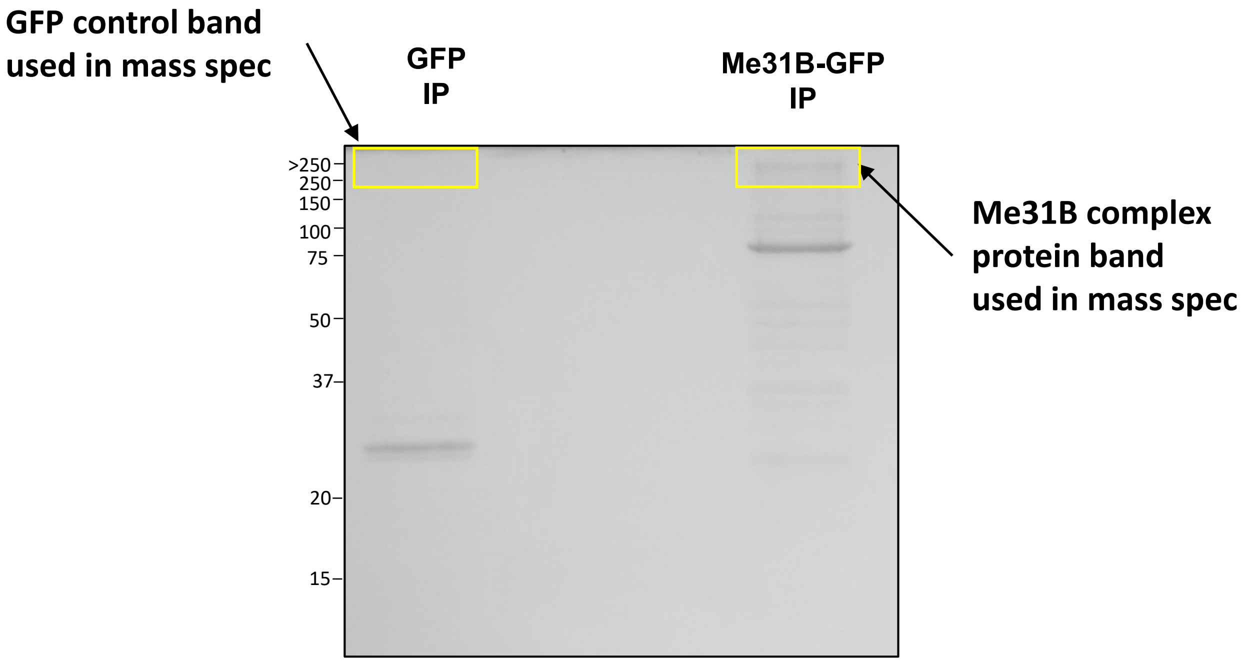

Me31B complex gel purification from 0 – 1 hour embryos (200 µl)

Supplementary Figure 2. Full-size blot images of main-text Figure 3C, 3D, and 3E.

Figure 3C. Full-size blot images with the cropped portion highlighted

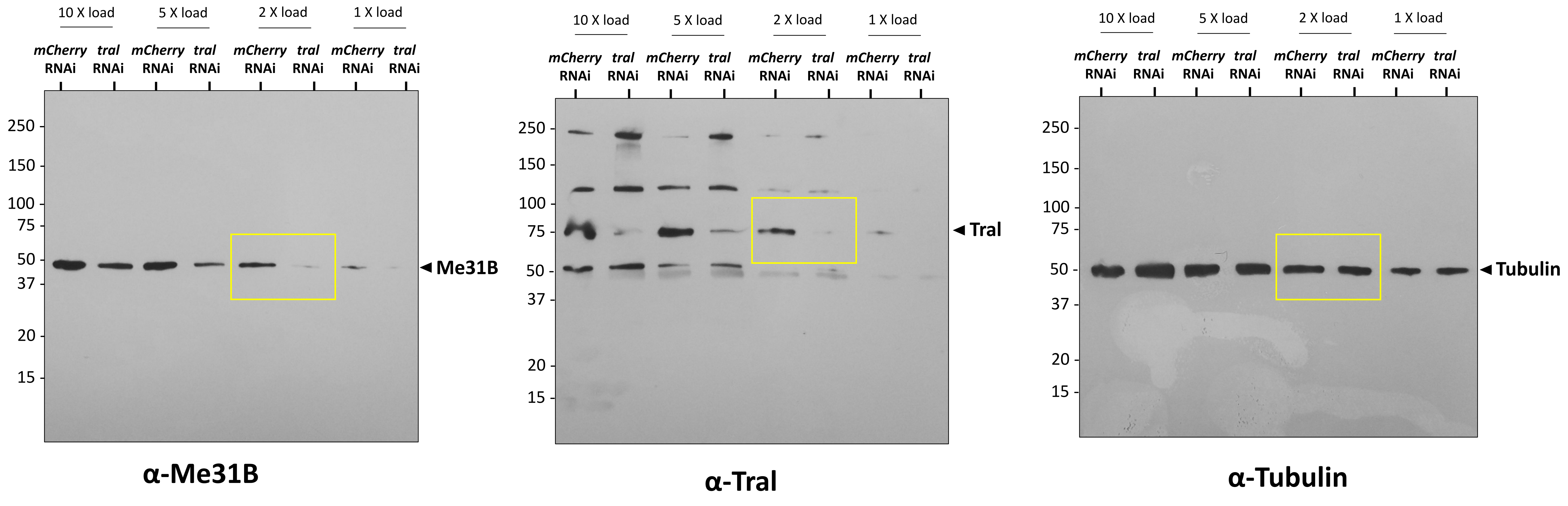

Figure 3D. Full-size blot images with the cropped portion highlighted

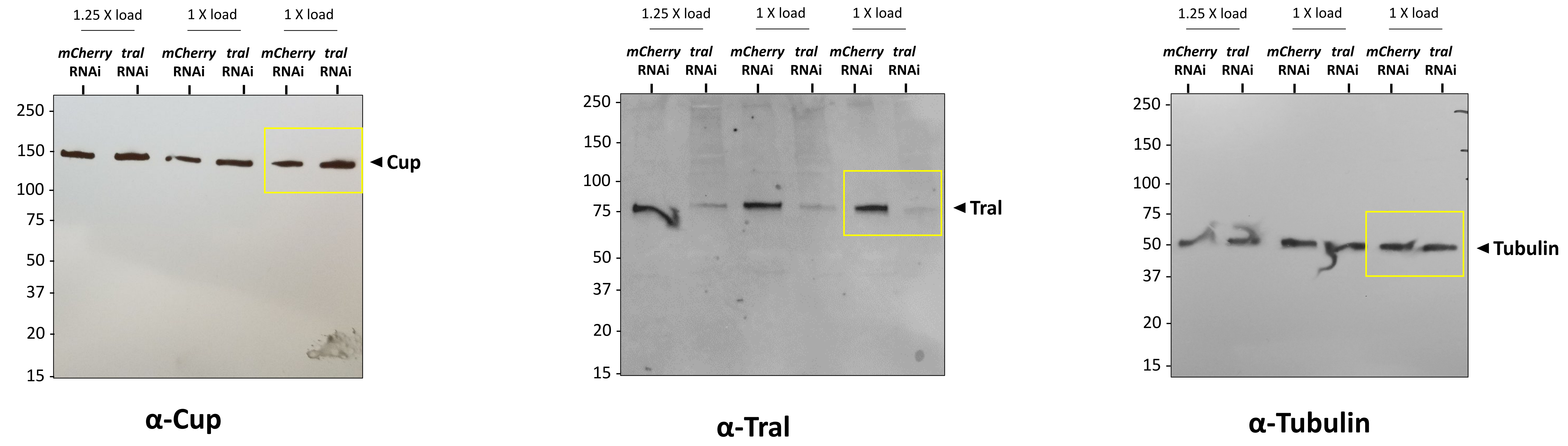

Figure 3E. Full-size blot images with the cropped portion highlighted

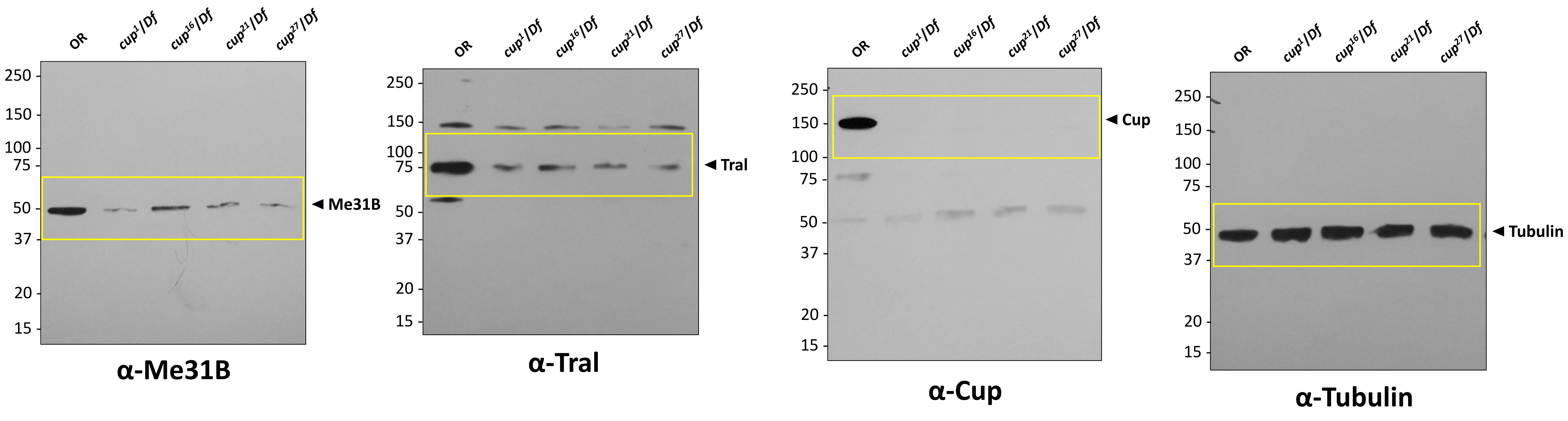

Supplementary Figure 3. Additional biological replicate images of main text Figure 3C, 3D, and 3E Western blots

Additional Biological Replicate 1: Anti-Tral, anti-Me31B, anti-Cup, and anti-α Tubulin Western blots in *tral* RNAi and mCherry RNAi ovaries

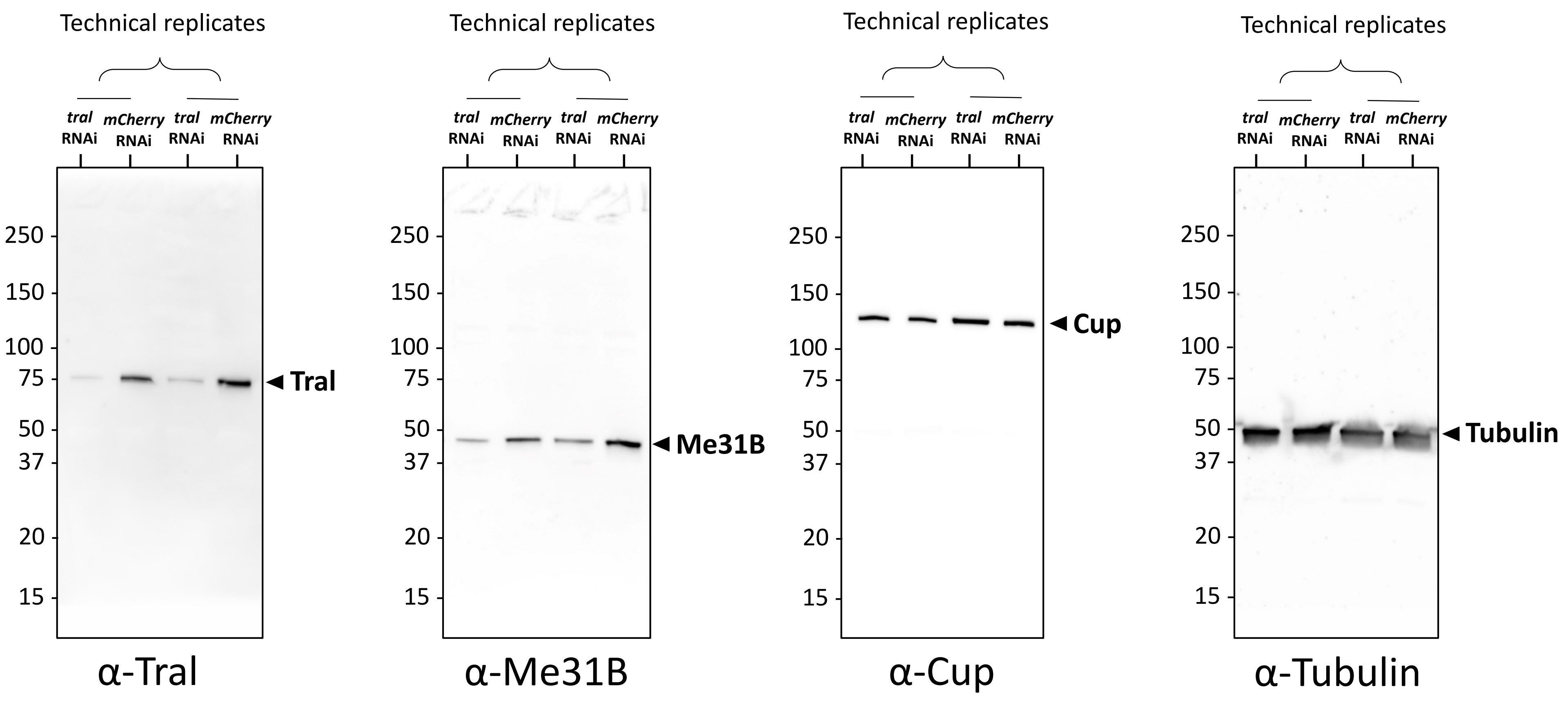

Additional Biological Replicate 2: Anti-Tral, anti-Me31B, anti-Cup, and anti-α Tubulin Western blots in *tral* RNAi and mCherry RNAi ovaries

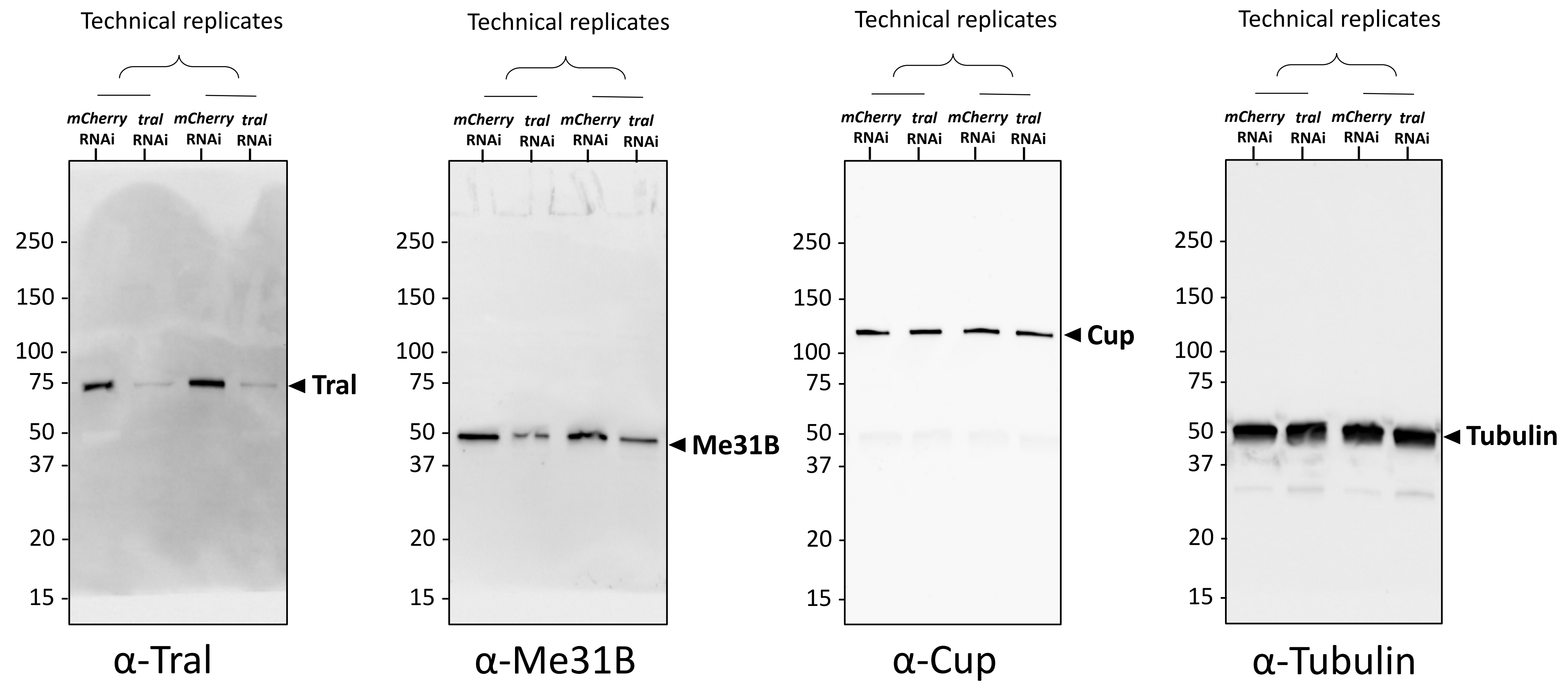

Additional Biological Replicate 1: Anti-Tral, anti-Me31B, anti-Cup, and anti-α Tubulin Western blots in *cup^{16}/Df* and OR ovaries

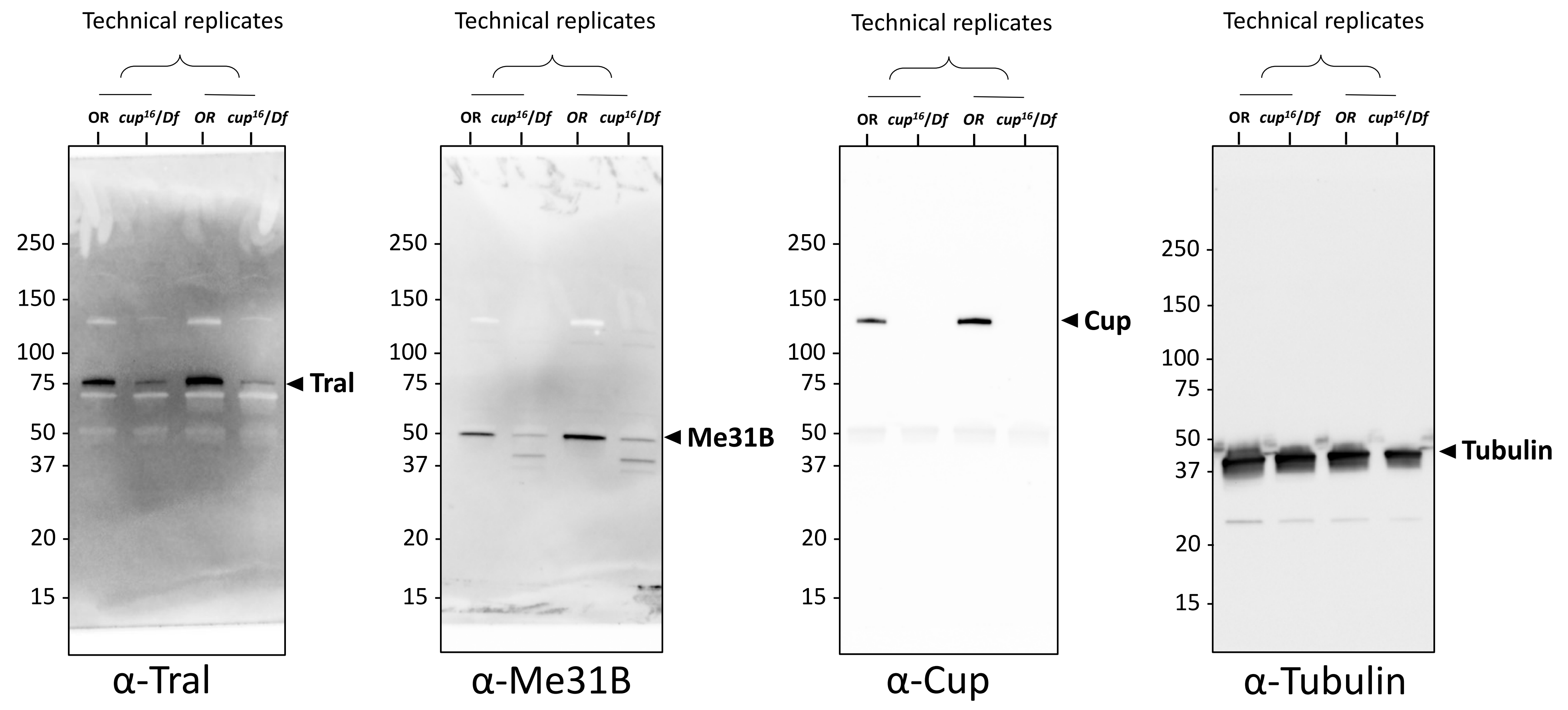

Additional Biological Replicate 2 and 3: Anti-Tral, anti-Me31B, anti-Cup, and anti-α Tubulin Western blots in *cup^{16}/Df* and OR ovaries

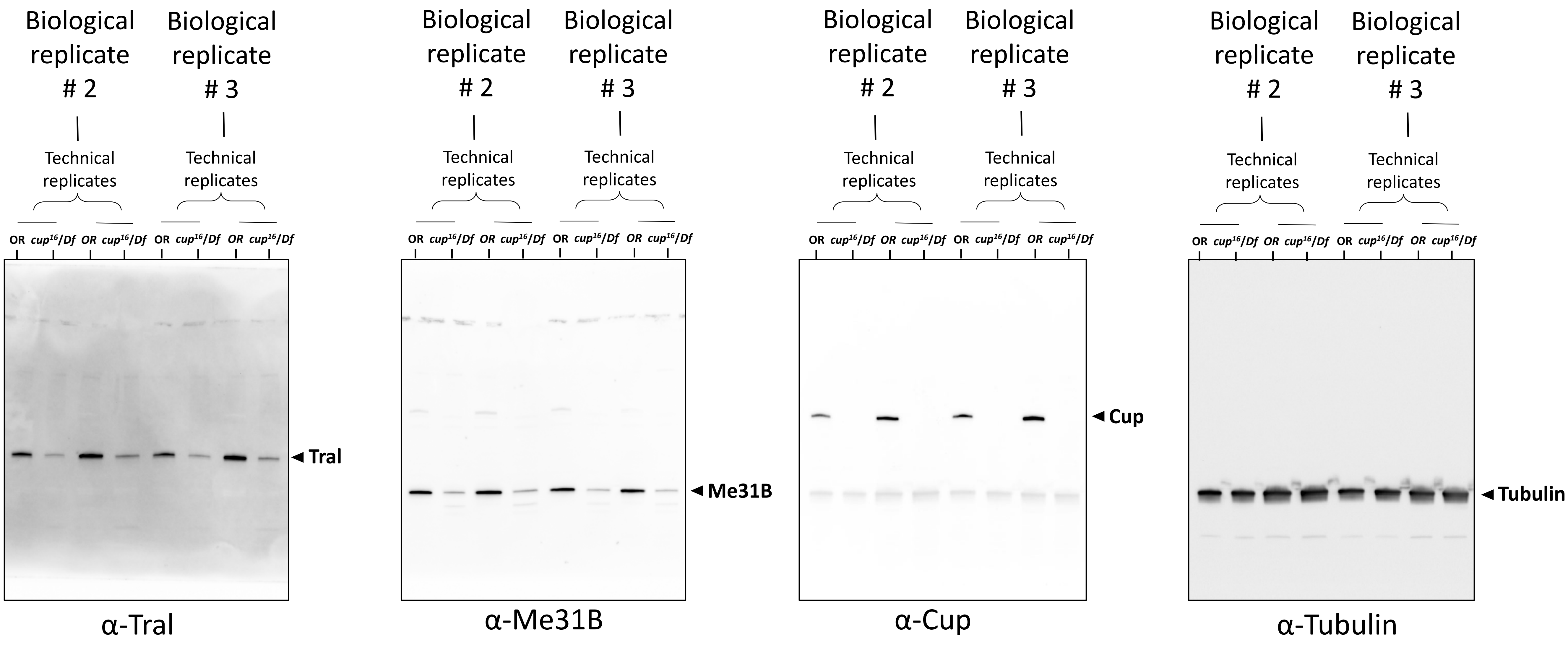

Supplementary Figure 4. Higher magnification image of Me31B-GFP and Vas-RFP localization in early embryo germ plasm.

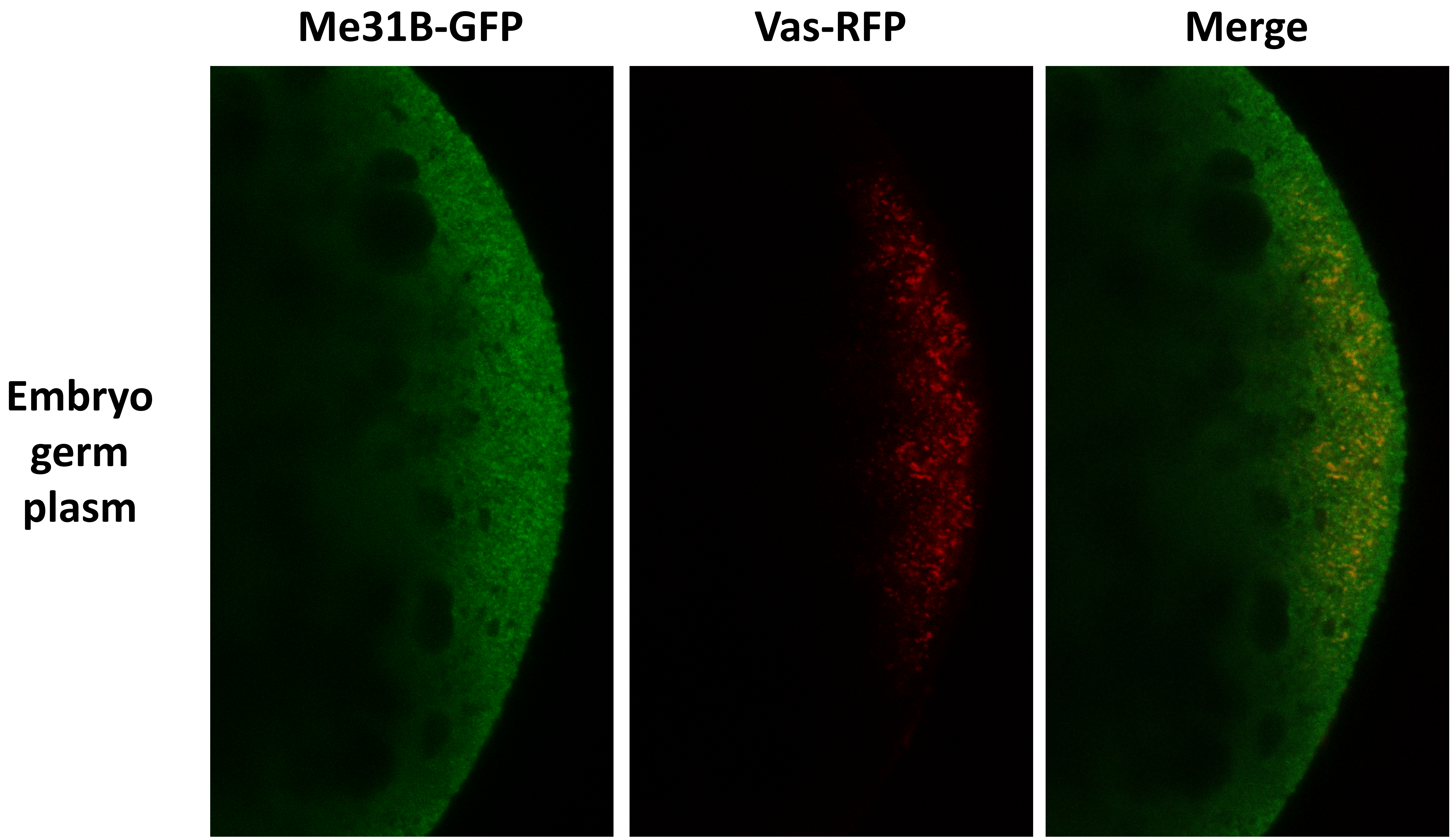

Supplementary Figure 5. Full-size blot images of main-text Figure 5B

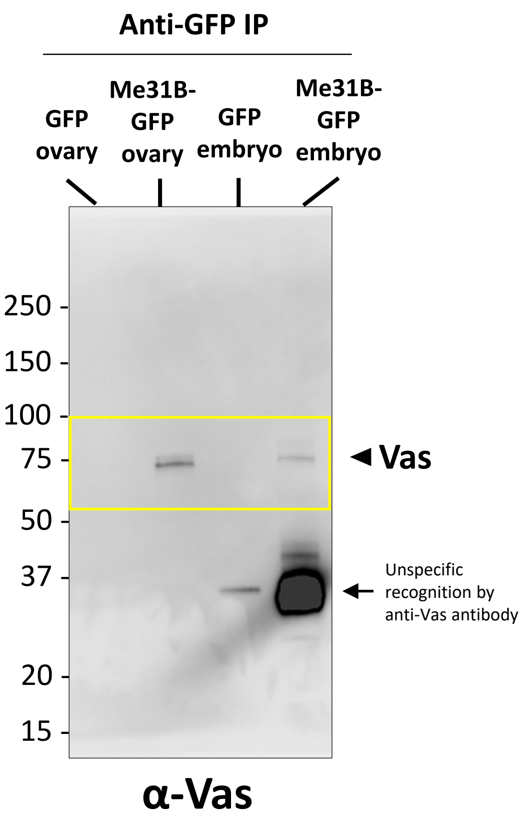

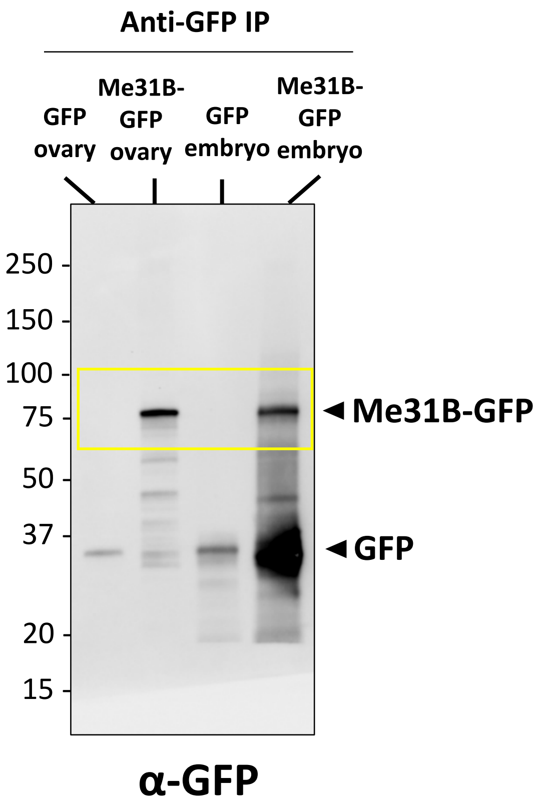

The highlighted rectangles indicate the cropped portion showed in the main text
